# Factors influencing the social acceptance of brain-computer interface technology among Chinese general public: an exploratory study

**DOI:** 10.3389/fnhum.2024.1423382

**Published:** 2024-10-30

**Authors:** RuiTong Xia, Shusheng Yang

**Affiliations:** School of Humanities and Foreign Languages, Qingdao University of Technology, Qingdao, Shandong, China

**Keywords:** technology, social acceptance, artificial intelligence, public attitudes, social influences

## Abstract

This study investigates the impact of social factors on public acceptance of brain-computer interface (BCI) technology within China's general population. As BCI emerges as a pivotal advancement in artificial intelligence and a cornerstone of Industry 5.0, understanding its societal reception is crucial. Utilizing data from the Psychological and Behavioral Study of Chinese Residents (*N* = 1,923), this research examines the roles of learning ability, age, health, social support, and socioeconomic status in BCI acceptance, alongside considerations of gender and the level of monthly household income. Multiple regression analysis via STATA-MP18 reveals that while health, socioeconomic status, social support, and learning ability significantly positively correlate with acceptance, and age presents an inverse relationship, gender and household income do not demonstrate a significant effect. Notably, the prominence of learning ability and social support as principal factors suggests targeted avenues for increasing BCI technology adoption. These findings refine the current understanding of technology acceptance and offer actionable insights for BCI policy and practical applications.

## 1 Introduction

Brain-computer interface (BCI) technology, a cutting-edge technology at the intersection of artificial intelligence and neuroscience, refers to the use of computer systems and other electronic devices to interact and communicate directly with brain activity (Wolpaw et al., 2000). It pushes the boundaries of traditional human-computer interaction by interpreting neural signals from the brain and allowing humans to interact with the external world without muscle involvement.

The concept of BCI technology was first introduced by Jacques Vidal in the 1970's—exploring the possibility of controlling external devices through brain waves, which marked the birth of the field of BCI research (Vidal and Bioengineering, 1973). As that research evolved, BCIs proved to have the potential for human applications (Dobelle et al., 1974; Kennedy and Bakay, 1998). As we enter the twenty-first century, BCI technology has been recognized for its potential in theoretical research (Eliasmith and Anderson, 2003; Graimann et al., 2010), application development (Berger et al., 2012; Hochberg et al., 2012; Ramos-Murguialday et al., 2013), and commercialization (Zhang et al., 2010). In particular, Neuralink's announcement to the public on January 28, 2024, that it has completed the first chip implantation in a human patient's brain, and Elon Musk's revelation that the patient appears to have made a full recovery, is further evidence of the great potential and achievements of BCI technology in medical applications.

While BCI technology continues to evolve in healthcare applications, it is also showing great potential in several areas of people's everyday practices, such as entertainment and socialization (Maiseli et al., 2023). For example, on March 20, 2024, Neuralink demonstrated in a live broadcast that the first human patient implanted with a brain-computer interface chip used BCI technology to play video games (Oi, 2024). Non-invasive BCI, especially EEG (electroencephalogram)—based technologies, even more so, have shown greater possibilities for widespread application and popularization in daily life due to their ease of operation, safety, and broad applicability (Zhang et al., 2019), such as the BCI game^1^ that has been widely used in recent years (Ahn et al., 2014). Taken together, advances in BCI technology have driven knowledge accumulation and technological innovation in cross-cutting fields, provided new tools and perspectives for understanding human consciousness and advancing social productivity, and had a profound impact on the functioning of human society.

Yet current societal attitudes toward BCI technology, in general, are manifested in two ways—on the one hand, the public has positive expectations about the potential of BCI technology to improve quality of life, particularly in terms of its ability to provide assistive tools for people with disabilities (Kögel et al., 2019; Schmid et al., 2021b); on the other hand, parts of the public have also shown concerns about the safety of individual consciousness and the potential for increased social inequality, as well as a caution about invasive interfaces (Schmid et al., 2021a). The public's acceptance of new technologies often directly influences their adoption rate and scope, a trend that holds for BCIs. As a form of social pressure, public sentiment significantly impacts investment decisions in the BCI field, thereby affecting its technological evolution (Kim, 2015). Furthermore, as BCIs alter how humans interact with the external world and are intricately linked to human bodily functions, issues of privacy, security, and ethics become paramount. The absence of clear ethical guidelines and legal standards necessitates that public attitudes and values be considered by governments and policymakers when setting regulations for BCI use, including conditions, modes, and scope of application (Livanis et al., 2024). Additionally, understanding the overall societal acceptance of BCIs is crucial for preempting and crafting policies that support equitable technological access and mitigate future social disparities.

To explore more specifically the social factors that influence public acceptance of BCI technology, this study begins by systematically reviewing existing literature on BCI acceptance and public attitudes, establishing a theoretical groundwork that informs our research questions and the identification of key variables. We then describe the methodology employed in the Psychology and Behavior Investigation of General Public in China (PBICR) project, detailing our data sample collection, characteristics, and research design, thereby clarifying our sources and variable settings. Through multiple regression analysis, we investigate the influence of seven factors—gender, age, monthly income, health status, social status, social support, and learning ability—on BCI acceptance among different demographics. This provides empirical insights into how these social factors correlate with technology acceptance. The paper concludes by addressing the study's limitations, suggesting strategies to enhance public acceptance of BCI, exploring avenues to foster broader approval, and offering perspectives on future research aimed at promoting a fair and sustainable technological ecosystem in the industry 5.0 era.

## 2 Literature review

BCI technology research has made significant progress in applied research in the past 5 years. The technical applications of BCIs are mainly categorized into invasive, semi-invasive, and non-invasive types, each targeting different application needs and technical challenges (Anupama et al., 2012). Current research focuses on improving the accuracy, stability, user interaction experience, and scenario-specific applications of brain-computer interface systems (Padfield et al., 2019). Researchers in the social sciences, on the other hand, have focused on the social impact of BCI technologies (Kinney-Lang et al., 2020; Chandler et al., 2022), ethical norms (Hildt, 2015; Ienca and Andorno, 2017), human-computer interactions (Holz et al., 2015; Rogel et al., 2020), and future applications (Grübler and Hildt, 2014). While researchers generally agree that BCI technology is still in its developmental stage, they also believe that it has the potential for a wide range of societal applications, while public attitudes have not yet been explored by researchers. Against this backdrop, this study will next conduct a literature review of the two areas of technology-related social acceptance research and public attitudes toward brain-computer interfaces research to lay the doctrinal groundwork for exploring the prospects for real-world pan-applications of BCI technology and the challenges it faces.

### 2.1 The social acceptance of technology

Social acceptance, also known as social recognition, is a classic issue in social psychology research. However, there is no clear definition of the concept, although it is often used in research in science and technology and social science. Williams summarized the psychological definitions of Acceptance by pointing out that there are four general agreements on Receptivity: “……taking or receiving something, as a pleasure, a satisfaction of a claim, or a duty; favorable reception, regard, or approval; assenting to or believing; and acceptableness” (Williams and Lynn, 2010). Some studies have also pointed out that acceptance does not only mean acceptance but also has a variety of meanings, such as support (Schade and Schlag, 2003). In recent years, the meaning of social acceptance has also been constructed, Wang has summarized social acceptance as the degree of acceptance of the social costs of a decision in his migration study (Wang et al., 2021). Wüstenhagen has deconstructed the concept of social acceptance concept into three dimensions: socio-political acceptance, community acceptance, and market acceptance (Wüstenhagen et al., 2007), and Ouellette categorizes social acceptance into policy acceptance and individual acceptance (Ouellette et al., 2017). The existing concepts of social acceptance are still not precisely defined, but what can be found is that all of them emphasize the influence of both objective social environment and subjective psychological factors.

In the field of social acceptance of new technologies, scholars often focus on the acceptance of different groups of people with different geographic regions, ages, genders, statuses, etc., gradually forming the discussion field of technology acceptance (Nadal et al., 2020). Acceptance, as opposed to adoption, is an important foundation for technology feasibility studies (Sollie, 2009).

In a point of proximity to traditional social acceptance research, scholars have also tended to explore the influencing factors affecting technology acceptance among audiences or the general public and attempted to fit models. The earliest model of technology acceptance was proposed by Fred Davis—the model consists mainly of two independent variables, perceived usefulness and perceived ease of use (Davis, 1989), to explore the degree of acceptance and willingness of users to accept information systems and technologies. After this, technology acceptance models adapted to the dynamics of more new technological developments such as TAM2 (Venkatesh and Davis, 2000), TAM3 (Venkatesh and Bala, 2008), and UTAUT (Venkatesh et al., 2003) have emerged, which are commonly used respectively in order to study the factors influencing technology acceptance in enterprise-level software and information systems, online services used by individuals over time, and a wide range of technologies and application domains, and the independent variables involved have become more diversified from the single variables of perceived usefulness and perceived ease of use. Based on perceived usefulness and perceived ease of use, social influence, cognitive instrument, gender, age, experience, voluntariness, performance expectancy, effort expectancy, community influence, and facilitating conditions multivariate independent variables are added to improve the explanation. However, the fact that social influence has often been ignored or weakened in past technology acceptance models and social norms have been used primarily to explain social influence has caused these models to be frequently challenged by focusing on the coverage of the meaning of social influence, and the validity of the variable settings (Malhotra and Galletta, 1999; Lee et al., 2006; Hubert et al., 2019). Therefore, it is common for contemporary social psychologists to incorporate new assessment models and try to include variables such as self-efficacy, attitudes toward learning, perceived difficulty, self-identity, and social support to improve the explanation of technology acceptance (Barnard et al., 2013), research on China's geographic environment also follows this trend (Mensah and Khan, 2024). Whereas, in recent years, AI acceptance has been a hot area in technology acceptance research, especially for AI in healthcare, variables on social norms and social influence as intentions that can positively predict acceptance behaviors (Gursoy et al., 2019) are often suggested to be included in the consideration of influencing factors on the acceptance of AI technology (Kelly et al., 2023). In general, existing studies have focused on the influence of age, gender, health status, self-efficacy, and social support variables regarding technology acceptance and AI acceptance. Based on this, part of our research questions is:

**RQ1:** How might subjective individual feelings, as identified in traditional technology acceptance models, influence public acceptance of emerging BCI technology?
**RQ2:** Considering the potential for widespread application, which social factors significantly influence BCI acceptance among diverse societal groups, and how might these factors challenge existing paradigms in technology acceptance research?

### 2.2 Public attitudes research toward brain-computer interface (BCI) technology

In research related to public attitudes toward BCI technology, early work focused on collecting public opinion through questionnaires to refine ethical norms for BCI (Nijboer et al., 2013). With the advancement of BCI technology, the focus of research has gradually shifted to both the audience's concern and demand for the use of BCI technology, even though the ethical issues are still of concern. At the same time, research methods have expanded from a single questionnaire to a combination of qualitative and quantitative (Pham et al., 2018)—in terms of research methodology, in-depth interviews often show that respondents, after being influenced by a variety of factors, may move from initial acceptance to rejection, which is at odds with mainstream research findings and exposes the limitations of qualitative studies with smaller samples and quantitative-only studies that fail to consider multiple influences in depth (Huggins et al., 2011; Vlek et al., 2012; Monasterio Astobiza et al., 2021).

However, existing research still mostly focuses on stakeholders in the medical field (Kögel et al., 2020; Monasterio Astobiza et al., 2021), such as BCI researchers and patients with amyotrophic lateral sclerosis (ALS), exploring their willingness to accept BCI technology and their personal feelings (Huggins et al., 2011; Vlek et al., 2012; Schicktanz et al., 2015). Scholars generally agree that different groups, especially those with disabilities, have positive attitudes toward BCI technology. Although a subset of research has begun to focus on the general public—exploring the discrepancy between ethical issues and public perceptions or generalized research on the acceptance of neurotechnical devices (NTDs)—these studies are still influenced by ethically relevant research that seeks to present representations and issues of the general public regarding the acceptance of BCI technology rather than exploring the factors that influence it (Schmid et al., 2021a). In addition, the sample distribution of relevant studies has mostly focused on Europe and North America [e.g., Germany (Sattler and Pietralla, 2022), Spain (Monasterio Astobiza et al., 2021), and Canada (Sample et al., 2020)], there is few discussion about Asian samples, especially Chinese samples. Thus, our final research question is:

**RQ3:** Within the current socio-cultural context in China, to what extent do non-ethical factors affect public acceptance of BCI technology?

## 3 Methodology

Based on the literature review, this study used a large representative national cross-sectional survey data (*N* = 1,923) from the Psychology and Behavior Investigation of Chinese Residents (PBICR)—to ensure the broad applicability of the results. The study design covered key social factors such as gender, age, level of monthly household Income, health, socioeconomic status, social support, and learning ability that have been identified in existing studies as potentially influencing technology acceptance.

In order to assess (test) the influence of these factors on BCI technology acceptance, we used STATA-MP18 software to conduct a multiple regression analysis—the questionnaire was set with the question “Would you be willing to adopt brain-computer interface technology, provided it is legal and technologically feasible to do so?” and used a 100-0 decreasing percentage scale to assess acceptance and as a dependent variable for correlation tests. This section will detail the specific methods of data collection, sample selection criteria, final validation of the independent variables, and testing methods.

### 3.1 Data sources and independent variable identification

The survey data used in this study came from the Psychology and Behavior Investigation of Chinese Residents (PBICR) project. This ongoing initiative, now in its fourth year, is a collaborative effort involving the Department of Social Medicine and Health Education at Peking University's School of Public Health, the Institute of Healthy Yangtze River Delta at Shanghai Jiaotong University, and Shandong Provincial Hospital. The PBICR project aims to examine the interrelations among various social, psychological, and behavioral variables to provide insights into the mental health status and related health behaviors of Chinese residents. It also tracks changes in the psychology and behaviors of the population over time.

The 2023 survey iteration specifically gathered data on societal attitudes toward current healthcare issues, including the COVID-19 pandemic, the granting of prescription rights to nurses, and the adoption of brain-computer interfaces (BCIs). An expert committee, comprising specialists in psychology, management, public health, and statistics, oversees the research design and survey methodology, ensuring robust and relevant data collection. The questionnaire, implemented through face-to-face field surveys conducted by a dedicated team of provincial coordinators and surveyors, is structured into seven modules: personal demographics, health status, family background, psychological scales, behavioral scales, other relevant scales, and sections addressing specific topical issues.

Our questionnaire leverages established international scales to assess mental health and has been tailored through systematic literature reviews and expert consultations to meet the unique needs of the Chinese population. Over the past 4 years, the content within each module has been continually enriched and optimized to capture a broader array of psychological and behavioral variables while maintaining an appropriate length for participant engagement. From 2020 to 2023, while updating and refining core variables, the PBICR has preserved ~30% of its longitudinal tracking variables, allowing for detailed trend analysis (Yibo et al., 2024; Yang et al., 2024). This study is based on the literature review and further analyzes and discusses the survey data from the brain-computer interface social acceptance study portion of the PBICR project.

This research used indicators across six domains: Basic personal and family information, Psychological dimensions, Social environment, Current events, and additional relevant scales. The scale development drew on established technology acceptance models, enhancing them with other relevant factors. Given that brain-computer interface (BCI) use in the broader population does not yet correspond with their actual experience—mostly observed in volunteers—the traditional Technology Acceptance Model (TAM) metrics such as Perceived Ease of Use (PU) and Perceived Usefulness (PEOU), along with the Unified Theory of Acceptance and Use of Technology (UTAUT)'s dimensions—Performance Expectancy (PE), Effort Expectancy (EE), and Facilitating Conditions (FC)—may not completely align with this study's context. This investigation, therefore, adapts these metrics to account for the social influences specific to AI and healthcare contexts, parsing out the ease of use into objective and subjective individual differences. It retains the UTAUT's Social Influence (SI) component, which assesses the extent to which an individual perceives that influential others endorse BCI usage. This dimension encompasses four scales: Family Communication and Support, Peer Support, Neighborhood Support, and Community Services Support. The overarching construct of Social Support was calculated using an equal-weight mean method across these scales.

In conjunction with Barnard's factors for the impact of technology acceptance, the scale introduced both self-efficacy, perceived difficulty, and attitudes toward learning variables and integrated these three variables into one independent variable, Learning Ability (LA). In addition, considering past research on technology acceptance, applications of the TAM have focused on the structural psychology aspects of the TAM, the study of perceived usefulness and perceived ease of use and the addition of additional structure to extend the TAM model, while ignoring social influences. This is particularly evident in the TAM2 and previous models (Furong and Xuelian, 2011). In the process of identifying the variables, the study also included Socioeconomic Status, health, and Level of Monthly Household Income variables as social influence level variables.

Finally, the independent variables of this study were Learning Ability (LA), Gender (GEN), Age, Health, Social Support (SS), Socioeconomic Status (SES), and Level of Monthly Household Income (LMHI). And this research identified the dependent variable as BCI technology acceptance (BCIA).

And Hypotheses (H) addressing a specific aspect of the literature review and research questions are:

**H1:** Learning ability (a combination of self-efficacy, perceived difficulty, and attitudes toward learning) positively influences the acceptance of BCI technology among the general population.
**H2:** Gender affects the acceptance of BCI technology in the general population group.
**H3:** Age has a negative correlation with BCI technology acceptance among general population groups.
**H4:** Health negatively affects the acceptance of BCI in the general population group.
**H5:** Social support positively affects the acceptance of BCI in the general population.
**H6:** Socioeconomic status (key social factors) is positively associated with BCI acceptance in the general population.
**H7:** The level of monthly household Income will positively impact the acceptance of BCI in the general population group.

### 3.2 Procedures for data collection

The questionnaire collected information related to LA, GEN, Age, Health, SS, SES, and LMHI of the participants to analyze the factors affecting the acceptance of BCI technology.

In the sampling stage of the questionnaire, the study determined the number of cities to be sampled based on the population base of Anhui Province, 8 cities were sampled using the random number table method, and the population of the final sampled cities accounted for 45.60% of the total population, Quota sampling is then carried out on the basis of the city's population base, with quota attributes of gender, household registration, and age, requiring a gender ratio of 1:1, a household registration ratio of ~1:1, and, in order to ensure a sufficiently objective ability to make judgments, an age requirement of 18 years of age or older, and a quota according to the age ratio of the local population.

In the questionnaire data collection stage, this study first set up a team of nearly 50 investigators across the province, and trained them in questionnaire ethics and questionnaire filling specifications, equipped with four investigator supervisors, and then, based on the results of the quota sampling in the questionnaire sampling stage, the investigators carried out face-to-face questionnaire surveys in the local area, and the investigator supervisors accompanied the supervision of the entire survey process. Finally, a total of 2,000 questionnaires were distributed and 1,924 questionnaires were recovered, of which 1,923 were valid questionnaires, with a recovery rate of 96.20% and a validity rate of 99.95%.

### 3.3 Data analysis

#### 3.3.1 Descriptive analysis

After data cleaning and preprocessing, the study processed a total of 1,923 respondents' questionnaires (*N* = 1,923), and the actual number of males and females of both sexes collected was 836 males and 1,087 females. Among them, the respective variables were set up in the following manner: BCIA, SS, and LA as a percentage, with scores proportional to the degree; gender according to the physiological significance, with 1 = Male and 2 = Female; and LMHI divided into 10 tiers from low to high, and SES divided into seven tiers from low to high. The variable health takes the VAS score from the EQ-5D scale, with 100 representing “best-perceived health” and 0 representing “worst-perceived health.” Respondents select the position on the scale that best represents their overall health on that day to obtain a self-assessed health score.

The characteristics of the data are shown in Table 1.

**Table 1:** Descriptive statistics.

**Variables**	**Obs**	**Mean**	**Std. dev**.	**Min**	**Max**	**p1**	**p99**	**Skew**.	**Kurt**.
LA	1,923	55.17	7.643	32.5	91.33	38	79.17	0.744	4.91
GEN	1,923	1.57	0.50	1	2	1	2	−0.26	1.07
AGE	1,923	39.43	17.57	18	94	18	79	0.52	2.16
Health	1,923	74.94	16.17	0	100	30	100	−0.81	3.85
SS	1,923	73.16	10.56	31.98	100	49.36	96.92	0.22	3.25
SES	1,923	4.09	1.23	1	7	1	7	0.09	2.90
LMHI	1,923	5.13	1.99	1	10	1	10	0.28	2.73
BCIA	1,923	54.36	24.88	1	99	4	96	−0.40	2.13

**Figure 1:**
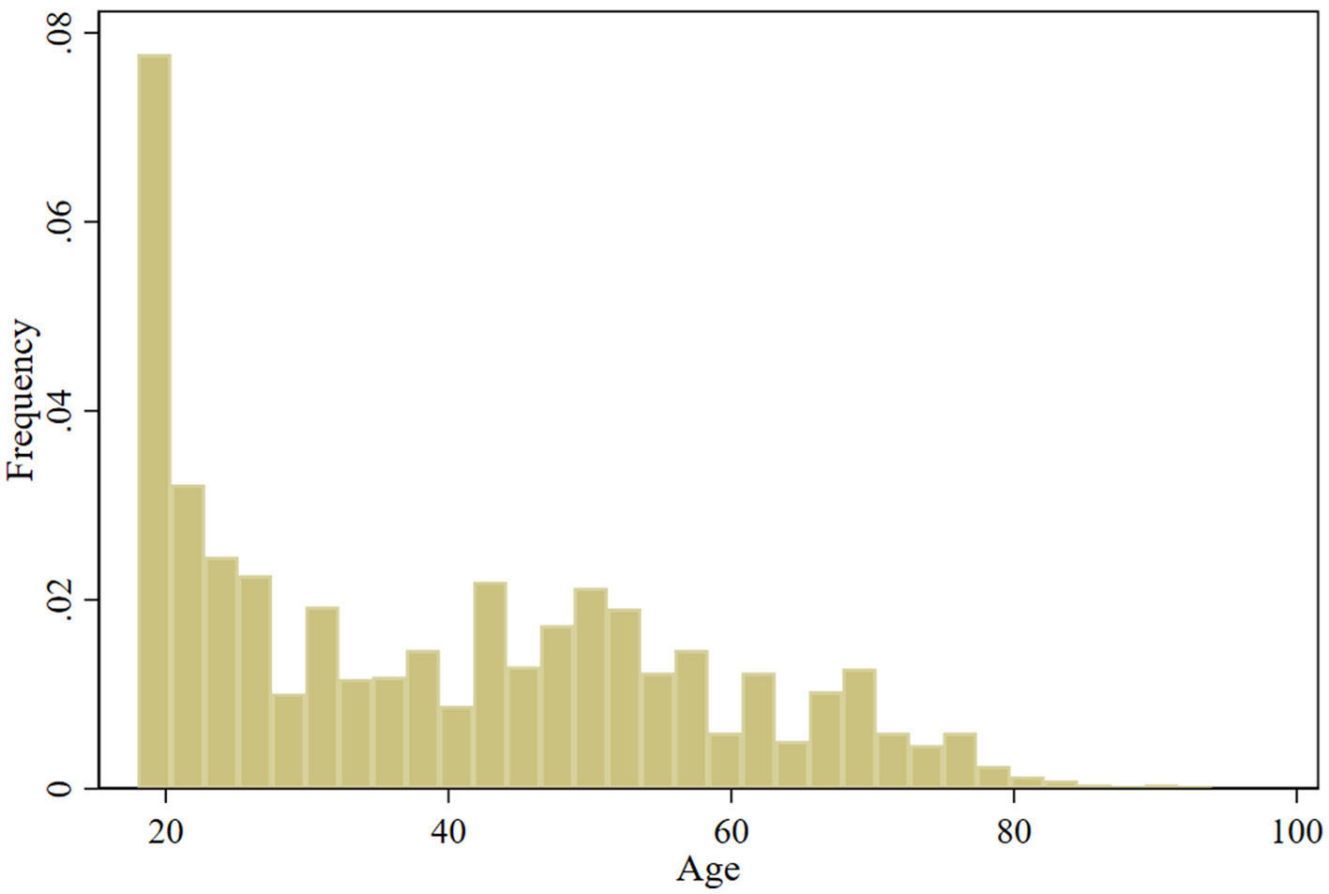
Histogram of age.

**Figure 2:**
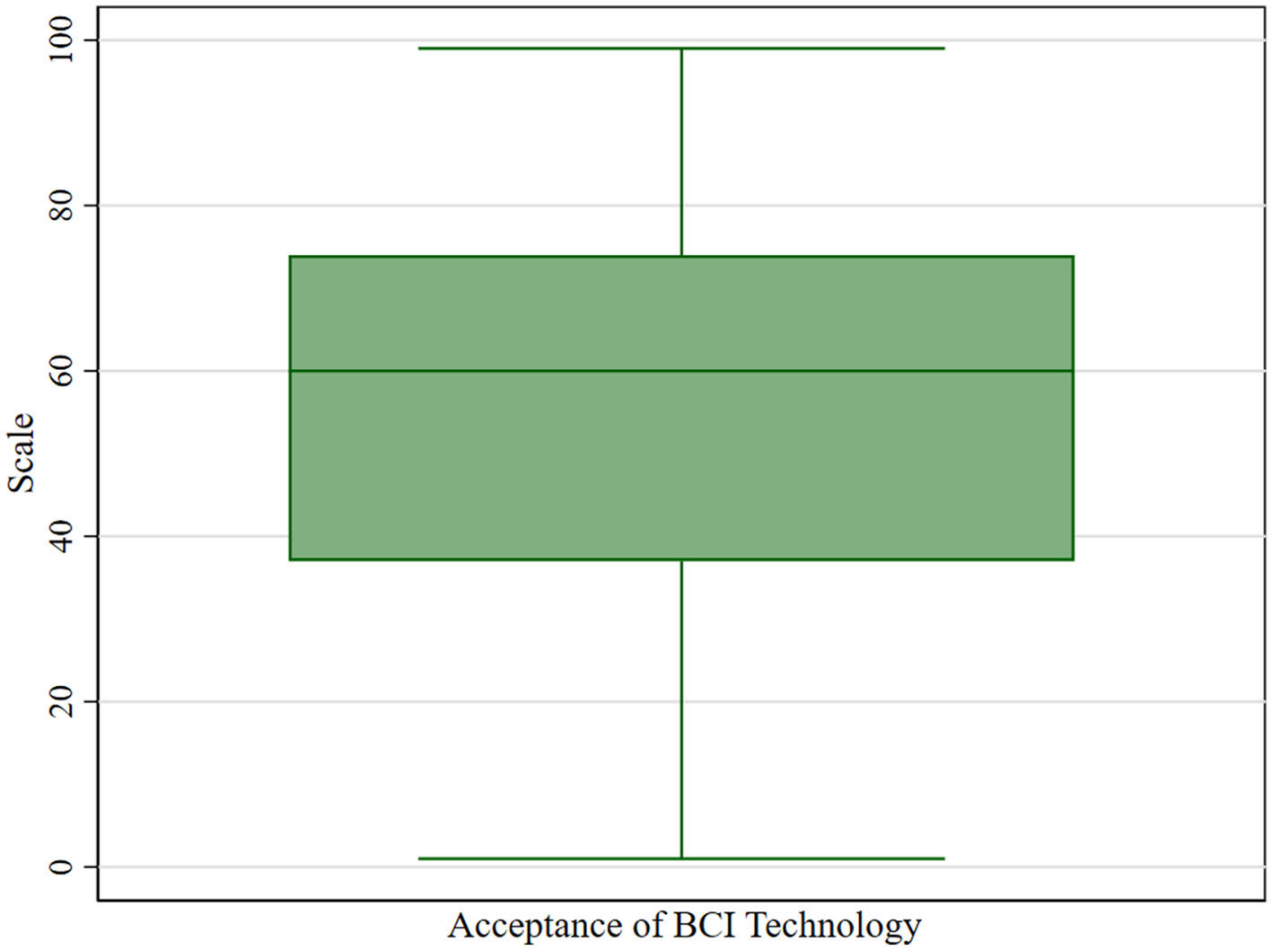
Box plots for BCIA.

**Figure 3:**
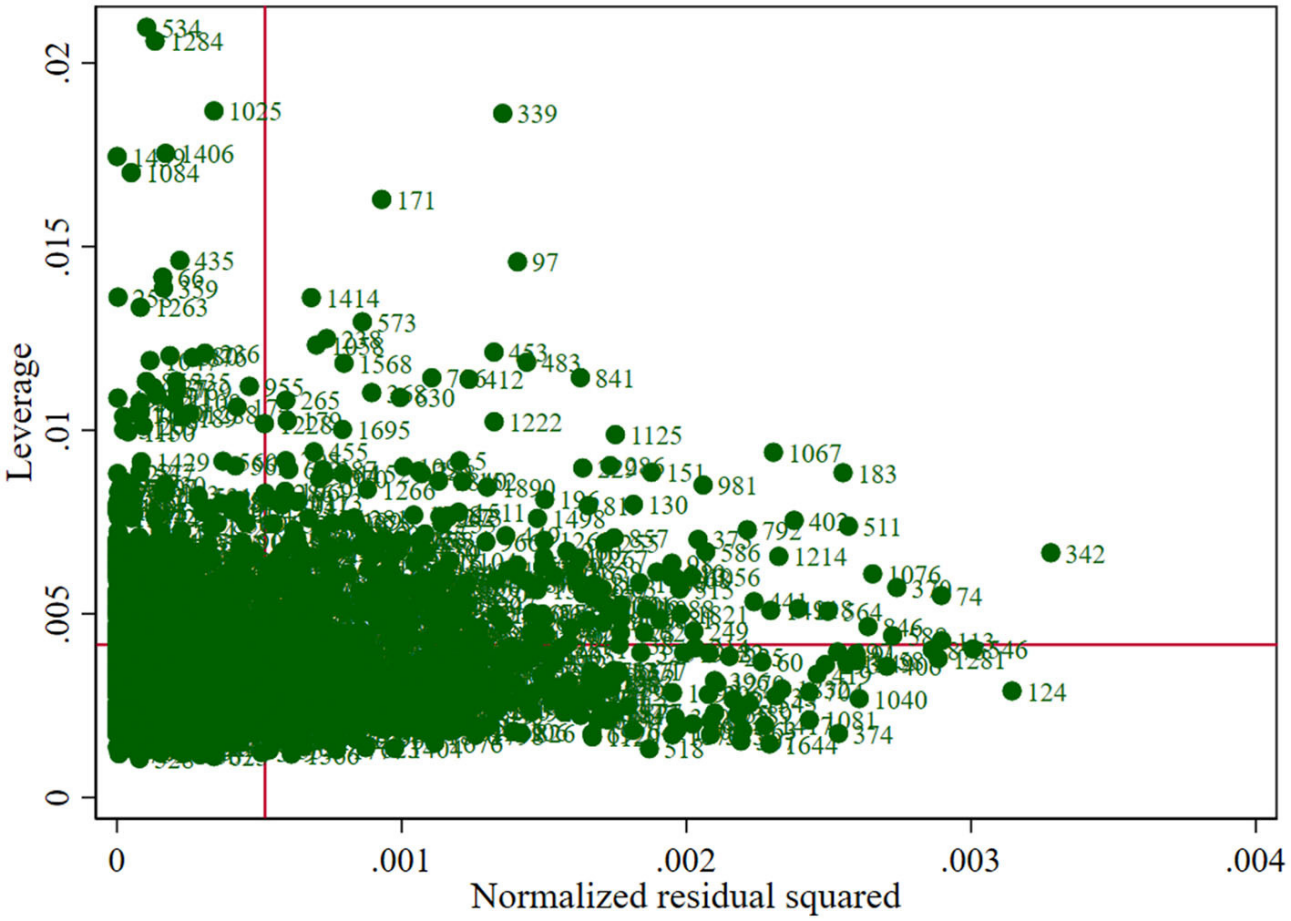
Residual plot with BCIA as the dependent variable and the remaining.

Therefore, all variables except for the GEN variable were continuous variables, and because BCIA is a continuous variable, it could be considered for linear regression analysis. However, due to the large sample size and the specific screening (excluding those under 18 years of age from answering) and correction (number correction for those between 18 and 22 years of age) that the study undertook to ensure that substantial parity in the number of people of all age levels was achieved despite ethical and cognitive level constraints, there may be potential outliers in the measurements of BCIA (as shown in Figure 1). According to the results of the box plots, residuals, and leverage values tests, there are indeed individual outliers in the dependent variable BCIA (as shown in Figures 2, 3 and Table 2) and individual high leverage values (as shown in Table 2, e.g., ID: 534, 1284) and observations with large squared standardized residuals (e.g., ID: 342), as well as individual outliers with large sessions of both (e.g., ID: 339).

**Table 2:** The leverage value checklist.

**BCIA**	**Coef**.	**St. err**.	***t*-value**	***p*-value**	**[95% Conf.]**	**[Interval]**	**Sig**.
LA	0.119	0.08	1.49	0.136	−0.038	0.277	
GEN	−0.187	1.134	−0.17	0.869	−2.411	2.036	
AGE	−0.17	0.036	−4.75	0	−0.24	−0.1	^***^
Health	0.088	0.038	2.34	0.019	0.014	0.162	^**^
SS	0.132	0.059	2.23	0.026	0.016	0.248	^**^
SES	1.748	0.495	3.53	0	0.777	2.719	^***^
LMHI	0.302	0.303	1.00	0.319	−0.293	0.897	
Constant	29.802	6.097	4.89	0	17.844	41.761	^***^
Mean dependent var	54.355			SD dependent var		24.877	
*R*-squared	0.042			Number of obs		1,923	
*F*-test	12.035			Prob > F		0.000	
Akaike crit. (AIC)	17,750.283			Bayesian crit. (BIC)		17,794.776	

^***^p < 0.0**1**.

^**^p < 0.0**5**.

^*^p < 0.1.

Therefore, to enhance the reliability of the analysis, we use robust estimation methods to estimate the linear regression coefficients to minimize the impact of outliers on the regression estimates. The following two conditions need to be met to perform robust linear regression analysis:

**Condition 1:** The dependent variable is a continuous variable. In this study, BCIA is a continuous variable, so this condition is satisfied.
**Condition 2:** Although linear regression analysis usually requires that the residuals follow a normal or approximately normal distribution, given the large sample size of this study, the central limit theorem can somewhat relax the assumption of normality of the residuals. In this study, histograms and Q-Q plots of the residuals will be used to test the normal distribution of the residuals after model fitting.

#### 3.3.2 Multivariate analysis and variable selection

In our previous analysis, we found individual outliers as well as high-leverage values in the study data. Since these outliers may distort the results of traditional correlation analysis and regression models, we used robust linear regression to improve the accuracy of the model. Therefore, the study directly used a one-factor robust linear regression model for causality analysis. In the robust linear regression analysis, age, monthly household income, health, socioeconomic status, social support, and learning ability variables showed significant correlations with BCIA (*p* < 0.05). However, the gender variable did not show statistical significance (*p* = 0.897, *p* > 0.1), and in subsequent multifactor regression models, we will consider the need to include gender in the model, depending on its theoretical significance and degree of contribution after controlling for other variables.

#### 3.3.3 Assessing multicollinearity and interaction among variables

In order to assess the case of multicollinearity in this study, we calculated the variance inflation factor (VIF) for each of the independent variables and found that all of the independent variables had a VIF value of < 5 (as shown in Table 3); therefore, in this study, each of the independent variables provides information that is independent of the others, and the model's explanations of the causality will not be compromised by over correlation between the independent variables.

**Table 3:** Independence test.

**Variable**	**VIF**	**1/VIF**
LA	1.210	0.826
GEN	1.020	0.979
AGE	1.270	0.785
Health	1.200	0.831
SS	1.260	0.794
SES	1.190	0.837
LMHI	1.170	0.856
Mean VIF	1.190	

We also conducted an interaction term test and did not find any significant interaction effects (*p*-values above 0.05 for all interaction terms), suggesting that the effects of the independent variables are additive rather than interactive. Therefore, there is no need to include interaction terms in the current regression model, and the simplified model is sufficient to explain the variation in BCIA.

#### 3.3.4 Analysis of multifactor regression efficacy for BCI acceptance

In the initial multiple regression analysis, we determined that the variables of AGE, LMHI, Health, SES, SS, and LA had clear causal relationships. After the initial model fit, gender still did not have a significant association, so variables out of GEN were considered for inclusion in the multifactor robust linear regression model to further explore their effects on BCIA. In the preliminary model fit (shown in Table 4), the individual effects of the level of monthly household Income (LHML) and learning ability (LA) did not show statistical significance (*p* > 0.05). Therefore, considering possible non-linear relationships, we transformed these variables, including calculating their quadratic terms, cubic terms, and inverses, and reintroduced these new forms of variables into the model for testing.

**Table 4:** Initial multifactor regression fit table.

**BCIA**	**Coef**.	**St. err**.	***t*-value**	***p*-value**	**[95% Conf.]**	**[Interval]**	**Sig**.
LA	0.115	0.085	1.34	0.18	−0.053	0.282	
AGE	−0.186	0.038	−4.90	0	−0.261	−0.112	^***^
Health	0.113	0.04	2.80	0.005	0.034	0.192	^***^
SS	0.157	0.063	2.49	0.013	0.033	0.282	^**^
SES	1.902	0.53	3.59	0	0.863	2.942	^***^
LMHI	0.31	0.324	0.96	0.339	−0.325	0.945	
Constant	26.964	6.097	4.42	0	15.006	38.922	^***^
Mean dependent var	54.355			SD dependent var		24.877	
*R*-squared	0.046			Number of obs		1,923	
*F*-test	15.451			Prob > F		0.000	

^***^p < 0.0**1**.

^**^p < 0.0**5**.

^*^p < 0.1.

The results of the multifactor regression fit are presented in Table 4.

After variable transformation and re-fitting, all transformed forms of LMHI remained insignificant (*p* > 0.05) in the model, hence the final decision to exclude it from the model. In contrast, the inverse transformation of learning ability (LA_x) showed statistical significance in the model (*p* < 0.05), suggesting that the relationship with BCIA may be non-linear. Accordingly, we constructed an ultimate multifactor robust linear regression model including age, health, social status, social support, and inverted transformed learning ability (LA_x). The results of the final model fit are shown in Table 5.

**Table 5:** Multifactor regression results.

**BCIA**	**Coef**.	**St. err**.	***t*-value**	***p*-value**	**[95% Conf.]**	**[Interval]**	**Sig**.
LA_x	−650.628	255.343	−2.55	0.011	−1,151.406	−149.849	^**^
AGE	−0.182	0.037	−4.95	0	−0.255	−0.11	^***^
Health	0.109	0.04	2.69	0.007	0.03	0.188	^***^
SS	0.151	0.062	2.42	0.016	0.029	0.273	^**^
SES	1.975	0.517	3.82	0	0.96	2.989	^***^
Constant	47.232	7.045	6.70	0	33.416	61.049	^***^
Mean dependent var	54.355			SD dependent var		24.877	
*R*-squared	0.048			Number of obs		1,923	
*F*-test	19.247			Prob > F		0.000	

^***^p < 0.0**1**.

^**^p < 0.0**5**.

^*^p < 0.1.

The overall statistical significance of the model was confirmed by the *F*-statistic and its corresponding *p*-value, which showed *F* = 19.25, *p* < 0.001, indicating that at least one of the predictor variables of the model statistically significantly predicted the dependent variable. In addition, every variable included in the final model was significantly correlated with BCIA (*p* < 0.05). The assessment of the goodness of fit of the model showed AICR = 1,551.14, BICR = 1,587.13, and deviance = 1,037,454.70, which are indicators that the selected model has a better fit and lower loss of information relative to the other models.

**Figure 4:**
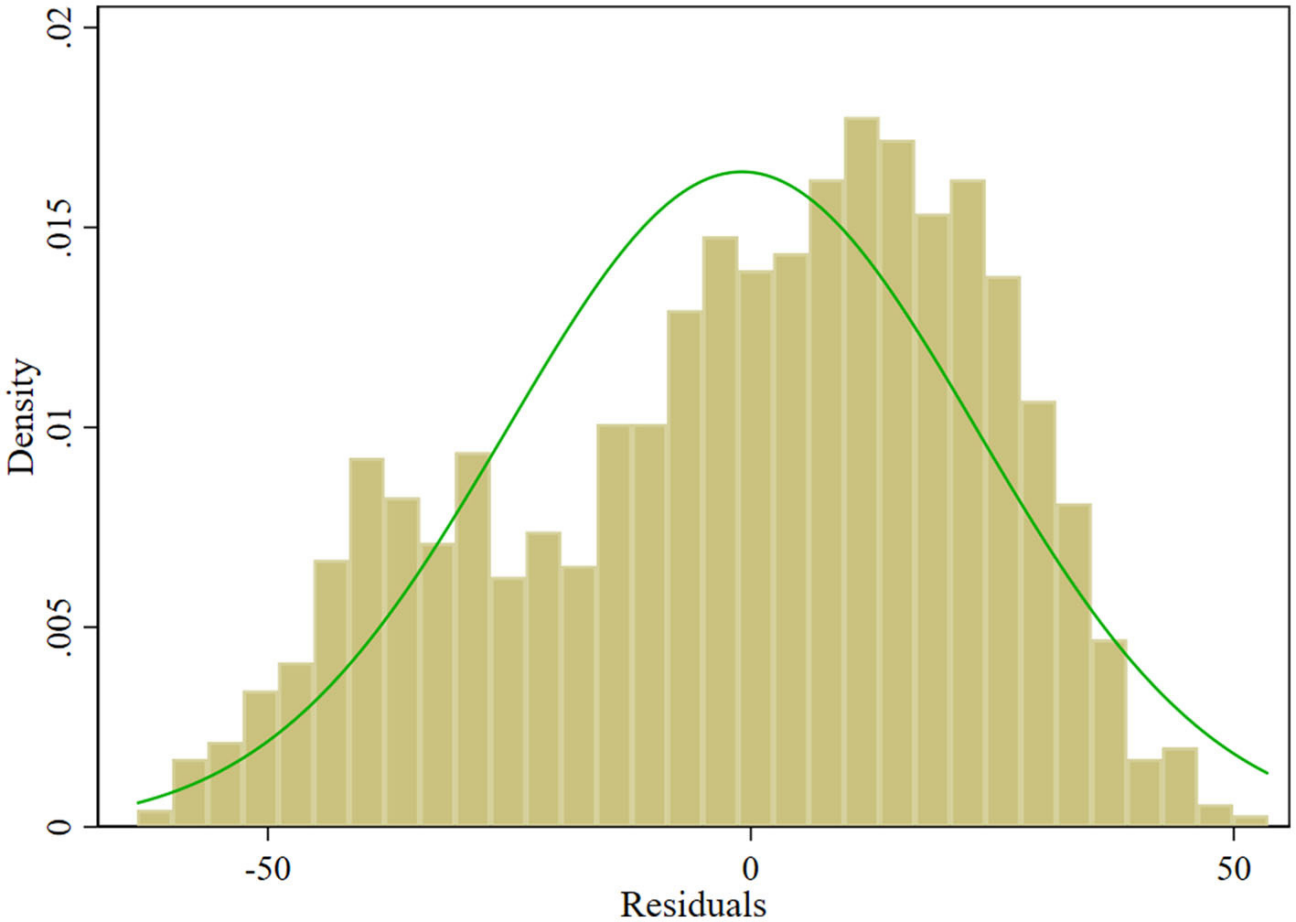
Histogram of residuals.

**Figure 5:**
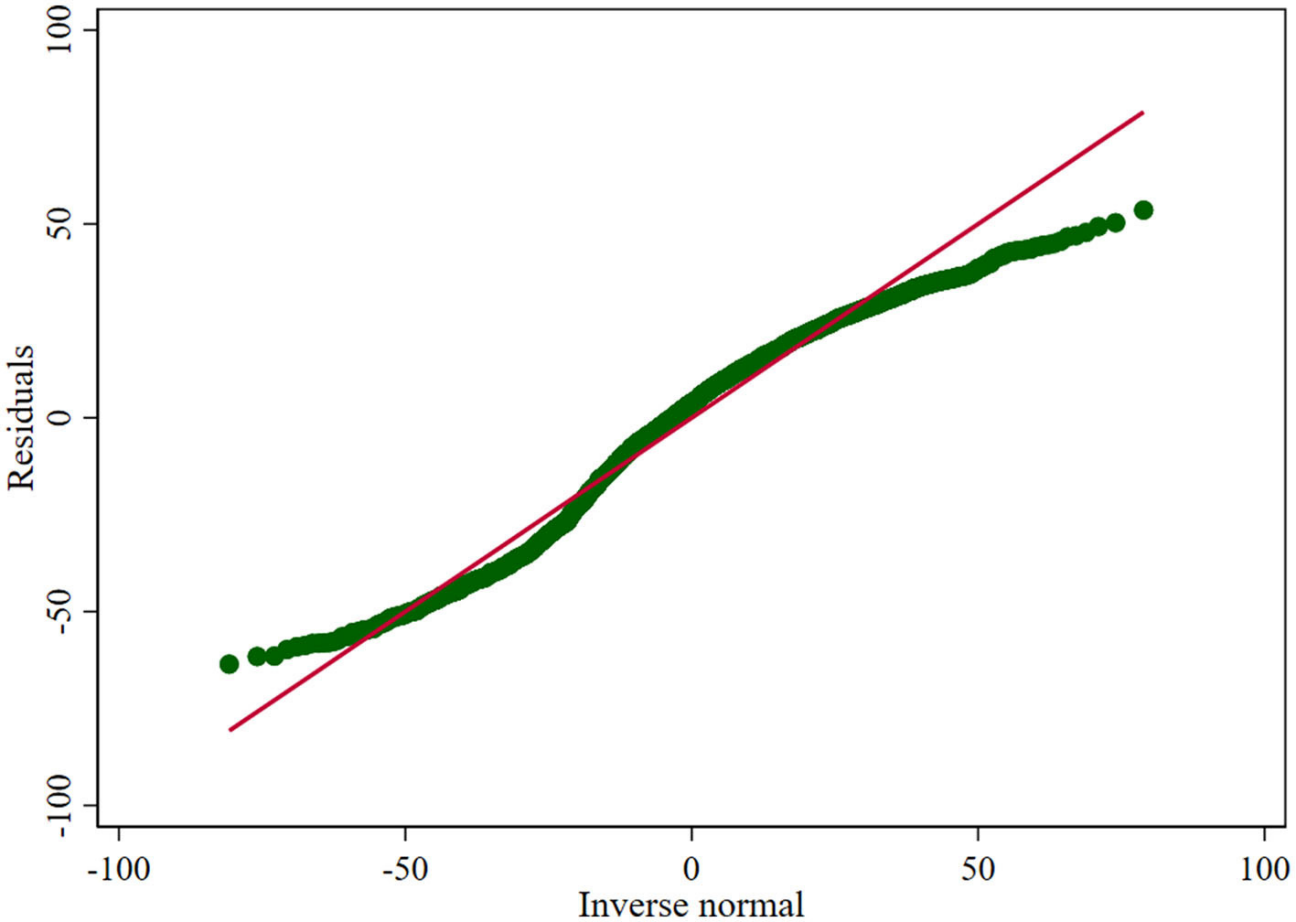
Residual Q-Q plot.

Specifically, the regression coefficients indicated that AGE was negatively correlated with BCIA, while Health, SES, SS, and LA were positively correlated with BCIA. Further analysis showed that among these variables, AGE and SES had a statistically more significant effect on BCIA than the other variables (*p* < 0.001). Although the combined explanatory power of these variables is limited, the overall significance of the model, as confirmed by the *F*-statistic (*F* = 19.25, *p* < 0.001), suggests that at least one of the factor variables is effective in explaining BCIA, and the fitted histogram of residuals with residual Q-Q plot indicate their approximate normal distribution (as shown in Figures 4, 5). Therefore, it can be concluded that these variables are statistically significant as factors.

## 4 Results

The results of the data analysis support the idea put forward in existing studies that social influences play an important role in the BCIA of the general public. Specifically, five variables-age (AGE), health (Health), socioeconomic status (SES), social support (SS), and learning ability (LA)-had statistically significant effects on the BCIA, suggesting that they are factors in understanding the variation in public acceptance.

And the analysis did not support H2, which proposed that gender would influence the acceptance of BCI technology among the general population, and H7, which predicted a positive impact of monthly household income on the acceptance of BCI technology within the general population.

The specific findings of the study are summarized below.

### 4.1 Learning ability and its correlation with BCI utilization

Learning ability consists of three dimensions: self-efficacy, perceived difficulty, and attitudes toward learning. The inverse of this variable was fitted to show a positive correlation with acceptance, with a coefficient of 255.3426, pulling up the percentage of positive correlation in the whole fitted model. It is worth mentioning that the learning difficulty dimension is mainly composed of stress scales, which validate the influence of stress factors on public attitudes toward brain-computer interface technology in previous studies (Atwal et al., 2021). And the self-efficacy and propensity toward learning dimensions, validate the findings of past studies that social factors have a significant impact on AI in healthcare (Gursoy et al., 2019).

### 4.2 Gender perspectives on BCI acceptance

Unlike existing studies or the UTAUT model, gender did not hold as a significant moderating variable in this study. In this study, the test of a one-way robust linear regression model verified that gender has no significant effect on BCIA and social acceptance in the current data, contrary to previous studies' findings. It shows that in the context of China's socio-cultural environment, the influence of gender is minimized, and the acceptance of brain-computer interfaces has the same generalizability in both male and female populations.

### 4.3 Age-related trends in BCI acceptance

Age has a significant influence relationship with a *p*-value infinitely close to 0 in the fitted model and a negative correlation with acceptance with a coefficient of −0.37. This finding is essentially the same as that found in previous studies, further validating the exact effect of age on willingness to accept BCI technology and demonstrating that the younger age group will have a higher level of acceptance of BCI technology.

### 4.4 Health as a factor in BCI usability

Health, a variable often mentioned in past studies of AI acceptance in healthcare (Ye et al., 2019; Dieter, 2021; Zarifis et al., 2021) was also verified to have an impact on the BCIA in the current study. Health status showed a positive correlation with a coefficient of 0.04 in the fitted model. This is contrary to the findings of previous stakeholder studies of BCI stakeholders that the worse the health status, the higher the willingness to use, demonstrating a change in the effect of influence in studies with the public as the subject.

### 4.5 The role of social support in the adoption of BCI

Social support is integrated into four dimensions: family communication and support, peer support, neighborhood support, and community services support, which covers the elements of social support comprehensively. The coefficient of social support factors in the fitted model is 0.062374, showing a positive correlation. The significant influence of social factors on AI in healthcare in previous studies was verified.

### 4.6 Socioeconomic status and its influence on BCI acceptance

The *p*-value in the model fitted by socioeconomic status is infinitely close to 0, which has a significant influence relationship, and its coefficient is 0.52, which shows a positive correlation. This finding highlights the considerable impact of socioeconomic factors on BCI acceptance, representing a novel contribution to this research and warranting further exploration in subsequent studies.

### 4.7 Level of monthly household income perspectives on BCI acceptance

Monthly household income is a demographic variable commonly assessed in sociological research and is thought to influence the market positioning of potential BCI technology consumers. Initial robustness regression analyses suggested a tentative positive correlation with BCI acceptance. However, this variable did not maintain significance in the multifactor model's later stages and was consequently excluded. Despite attempts to model its relationship by including quadratic, cubic, and inverse transformations, no significant association was observed upon multiple iterations. The implications of household income for the commercial viability and consumer adoption of BCI technologies remain inconclusive, highlighting an area for further empirical exploration.

## 5 Conclusion

In summary, this research identifies key determinants affecting the societal acceptance of brain-computer interface (BCI) technology, with age, health, socioeconomic status, social support, and learning ability emerging as significant influencers. Health, socioeconomic status, social support, and learning ability exhibit a positive correlation with BCI acceptance, while a reverse relationship is observed with age. Particularly, age and socioeconomic status exert the most profound effects. The investigation also indicates that gender does not have a notable impact on BCI acceptance within the Chinese population. Although household income positively relates to acceptance, its effect does not reach a level of significance warranting inclusion in the final model.

Thus, the key to increasing public acceptance of BCI technologies lies in enhancing social support and improving individual learning capabilities. Relevant stakeholders can conduct educational activities to increase the popularity of BCI knowledge, establish support communities to facilitate communication among users and provide targeted training to enhance the ability to utilize different types of BCI technologies. These initiatives will assist the public in overcoming concerns about new technologies and promote the widespread acceptance, use, and improvement of BCI technologies.

Taken together, this study breaks away from the ethically dominant orientation of past research by starting from an interdisciplinary perspective and designing with both the demographic variables factored into traditional social psychology research and integrating with technology acceptance theory. Through a multidimensional analysis of the acceptance of BCI technology, the study both verifies the applicability and problems of the influencing factors of traditional technology acceptance theories in the field of BCI technology and further expands the possible influence of socio-demographic characteristics on technology acceptance. Based on a large sample of multi-age and multi-region levels in China, the study breaks through the scope of previous studies that mainly focused on the attitudes of brain-computer interface stakeholders and focuses on the social and cultural context of China, filling the gap in the existing literature on the acceptance of BCIs in non-Western cultural contexts. The question of the influencing factors of BCI acceptance at the social level is addressed, laying the foundation of social investigation for developing and popularizing brain-computer interface technology.

## 6 Limitations and future work

The study of the public acceptance of brain-computer interface (BCI) is of great significance for both its own technological development and the formulation of related policies, as well as for the future development of society. Its public acceptance not only determines the speed of its promotion and application as a health technology (Lupton, 2017), but also directly affects the policy formulation and the construction of social ethical framework (OECD, 2017).

While this study provides significant insights into the public acceptance of brain-computer interface (BCI) technology, it is subject to certain limitations. The technology has not yet reached widespread adoption among the general public, and our dataset primarily comprises volunteers, which may limit the generalizability of our findings to practical use scenarios. Additionally, the acceptance of BCIs in our study was evaluated as a unified category, predominantly representing invasive types available on the commercial market. This approach may not fully capture the complexities of public acceptance for different types of BCI technologies, as indicated by the lower *R*-squared value (0.0447) of the model. This suggests that other significant influences specific to BCI technology were possibly overlooked.

Despite these limitations, this study reveals that age, health, social status, social support, and learning ability have significant effects on BCIA, whose significance is not limited by the explanatory power of the model. These findings pave the way for future research to explore comparative analyses across different demographic groups, including users and non-users but stakeholders. In particular, BCI technology offers a crucial means of overcoming physical limitations, significantly enhancing social competence for those with severe disabilities and sensory impairments, where such advancements are most impactful (Klein et al., 2016). In contrast, non-disabled populations, including technology enthusiasts and gamers, typically face fewer barriers to mobility and training. These groups often prioritize enhancements in the accessibility and performance reliability of BCI technology, aiming to broaden its applications across various settings (Karikari and Koshechkin, 2023). Given the varied urgency and perceived usefulness of BCI across these different populations, future research could delve into comparative studies across these diverse groups, examining the specific needs and acceptance levels of each demographic.

Besides, past research indicates that the adoption rates and acceptance of BCI technology can vary based on its type—whether invasive, semi-invasive, or non-invasive—each differing significantly in terms of health risks and utility (Kögel et al., 2019; Eldawlatly, 2024). Invasive BCIs, requiring craniotomy, offer clearer signals and are primarily used in medical settings due to their high health risks but significant benefits in terms of enhancing personal independence. Non-invasive BCIs, which do not require surgery, present the lowest health risks but yield the most ambiguous signals, making them suitable for everyday life and entertainment. Semi-invasive BCIs represent a middle ground. Considering how each type influences public acceptance is key for tailoring BCI development to meet societal needs and minimize risks. Therefore, future studies could focus on the specific applications of these technologies and their implications for user acceptance in different life contexts, and ensure that these technologies align with ethical standards and public expectations.

Overall, these concerns will better help policymakers and technology developers increase the acceptance and popularization of the technology. It would also align future advancements in BCI technology with societal needs and expectations, promoting equitable and responsible technological integration and forming a more transparent and responsible environment for technological development, thereby promoting the true benefit of BCI technology to the public.

## Data Availability

The raw data supporting the conclusions of this article will be made available by the authors, without undue reservation.

## Footnotes

^1^For instance, in the BCI game developed by Mentalista (Paris, France) for the 2016 European Football Championship, two players were asked to score against each other by moving a ball toward the opposite player's cage by concentrating (source from: https://mentalista.fr/foot).
